# 

**Published:** 1871

**Authors:** 


### CONTENTS:

ORIGINAL COMMUNICATIONS:

ART. I. A Few Practical Hints on Sponge Tents. By Philip Adolphus, M.D. .... 577

ART. II. On Abdominal or Ileo Typhus. By Carl Proegler, M.D. .... 585

ART. III. Surgical Clinic of Prof. Gunn. Reported by Dr. O'Brien... 593

ART. IV. Transfusion of Blood. By Carl Proegler, M.D. .... 595

ART. V. Death: Legal and Actual. By I. N. Danforth, M.D. .... 600

CORRESPONDENCE:

Modern Tendency of Homœopathy, 608

SELECTIONS:
Management of the Obstetrical Forceps. By C. C. P. Clark, M.D. .... 623

EDITORS' BOOK TABLE .... 644

EDITORIAL .... 669

AN APPEAL to the Alumni and Friends of Rush Medical College, for aid to assist in its rebuilding .... 674

STATEMENT OF OFFICIAL ACTION, with amounts contributed for Relief of Physicians, sufferers by the late fire, 676

LIST OF PHYSICIANS, sufferers by the fire, 685

NOTICES .... 689

